# Addictive behaviors: decades of research, but still so many questions!

**DOI:** 10.3389/fpsyg.2024.1485118

**Published:** 2024-11-06

**Authors:** Salvatore Campanella

**Affiliations:** Laboratory of Medical Psychology and Addiction, CHU Brugmann, ULB Neuroscience Institute, Université Libre de Bruxelles, Brussels, Belgium

**Keywords:** addictive behaviors, compulsion, dependence, mechanisms, biomarkers, treatment

Dominated by a “market capitalism”, i.e., an economic model promoting the buying and selling of goods and services, we nowadays live in a competitive, stressfull and mass consumption society, where people increasingly exhibit behaviors aimed at satisfying personal and material desires (Pérez and Esposito, 2010). Throughout their life, many people will engage in “excessive consumption acts”, but only some of them will move toward unrestrictive, unrestrained, uncontrolled, maladaptive, i.e., addictive, behaviors. Repeating an activity despite the explicitly stated desire to stop it due to its harmful individual and social consequences is the hallmark of an addictive behavior (Santangelo et al., 2022).

Decades of research have tried to understand which mechanisms trigger the onset as well as the long-term maintenance of such behaviors. Why and how can an arbitrary activity, such as buying clothes, eating food, watching TV series, drinking alcohol or gambling on sport events, become so central in a person life, and so compulsive that it will overtake all other activities? Classic addictions to drugs or substances may be understood by means of their reinforcing properties on specific brain networks. Indeed, despite their various molecular targets, addictive substances (such as alcohol, cocaine, heroin, tobacco) will act, by increasing dopamine level, on a common biological pathway, i.e., the mesolimbic circuit, and more precisely, in the nucleus accumbens of the ventral striatum, resulting in an interoceptive feeling of pleasure (reward) (Di Chiara and Imperato, 1998). Repeated exposure to the substance will promote the encoding of the rewarding properties of this event, acting therefore as a reinforcement learning signal, increasing the incentive salience of this reward and stimulating motor action (Berridge and Robinson, 1998). Transition from “simple impulses to consume” toward “compulsive behaviors” appears to be linked to a dysfunctional (pre)frontal control system, implicated in future-oriented processes and regulating current actions in relation to long-term goal-directed motivations (Robinson and Berridge, 2003). Overall, a substance-related addictive behavior may be seen as a reinforced-learned automatic response (a habit) to a highly salient activity, resisting to devaluation as well as to goal-inappropriateness (see Lüscher et al., 2020 for a review).

Nowadays, the mechanisms behind how such habits are learned and operate remain a topic of ongoing research, with controversies regarding the exact contribution of (overestimated) habitual processes vs. (underestimated) contextual goal-directed processes in the long-term maintenance of maladaptive behaviors (Bouton, 2021; Buabang et al., 2021). Also, if we can clearly imagine how those substances, acting as neurotoxic agents that kill neurons (Singh and Verma, 2020), could impact various brain systems—such as contributing to the hypo-activity of control regions in the frontal cortex—the situation is even less clear when we consider *non-substance-related* addictive behaviors. Indeed, in the absence of any ingestion of a neurotoxic agent, how can we understand that behaviors such as playing video games, gambling, watching TV or social media use may become “addictive”, and represent (mainly among youths) a significant public health concern? Of course, all these behaviors are “rewarding”, therefore also creating through the dopamine mesolimbic system highly salient cues hijacking attention and stimulating compulsive action (Vidal and Meshi, 2023). But how can we explain the transition to compulsion in the absence of any neurotoxic effect?

Several explicative “*individual risk factors*” for the development of addictive behaviors from adolescence to adulthood have been proposed, such as consumption motives (social, pleasure, coping…), family history (genetic part as well as education), associated conduct disorders, inter-individual differences in cognitive control or emotional regulation skills, personality traits (impulsivity, sensation seeking, risk taking…) or gender (Petit et al., 2013). Some evidence also points to *implicit cognitions*, i.e., unconscious learned associations in memory (“When I feel bad, if I drink, I feel better”), which may explain why people can engage in behaviors they know being life threatening (Stacy and Wiers, 2010 for a review). Altogether, all of this makes the story particularly complex, and, despite decades of research, it still triggers more questions than responses. Addictive behaviors related or not to a substance present a strong common neurobiological link (Grant et al., 2006), but also some specific patterns of response to rewarding cues (Dubuson et al., 2023). Given their high co-occurrence (Burleigh et al., 2019) and the rise in polydrug use (Crummy et al., 2020), further research is highly needed for improving the understanding of the mechanisms triggering the onset of such maladaptive behaviors (maybe by promoting more translational research going from animal to human research), as well as prevention and treatment strategies. Prevention is particularly difficult to achieve, as, due to the huge number of potential contributive factors, it is impossible to give a clear recipe to apply. Studies that will help to define *profiles* of high-risk people for developing addictive behaviors remain therefore of the highest importance at the fundamental and clinical levels, as moreover, we are all aware that the impact of current conventional proposed treatments (based on detoxification, psychotherapy and anti-craving medication), is modest. Aggregate substance use disorder (SUD) remission (abstinence) rates were 10–15% for any given year (Fleury et al., 2016), pointing to the urgent need to find complementary and effective add-on tools, not only to globally enhance quality of care, but also to reduce treatment gap (only a small proportion—around 10%—of adults with SUD seeks treatment) (Knopf, 2024).

The last decades have seen the emergence of various forms of add-on tools. New dimensions of *psychological interventions* were proposed and tested, such as the use of mindfulness (Korecki et al., 2020); of eye movement desensitization and reprocessing (EMDR; Carletto et al., 2018); of cognitive behavioral therapy (CBT; Ray et al., 2020); of cognitive bias modification programs (CBM; Manning et al., 2019); or of new technologies promoting remote or automated interventions through web-based or smartphone-based apps (Ferreri et al., 2018). *Neurocognitive models* also described two opposite systems underlying addictive behaviors: an abnormal bottom-up (impulsive, limbic) system generating implicit attentional biases (increased attention specifically related to alcohol cues), a “wanting” (craving, i.e., desire to drink) behavior and an automatic approach tendency, which cannot be “regulated” by a weakened reflective/executive (control, prefrontal) system that cannot inhibit approach tendencies (Wiers et al., 2007). On this basis, many studies have sought to promote abstinence by decreasing attentional biases and/or increasing inhibitory skills, through cognitive training (MacLean et al., 2018; Verdejo-Garcia et al., 2023), by using neuromodulation tools such as TMS (Antonelli et al., 2021) or tDCS (Lupi et al., 2017); or even by combining cognitive training programs with neuromodulation tools (Dubuson et al., 2023). Tools such as neurofeedback (Russo et al., 2023) or virtual reality (Tsamitros et al., 2021) were also used for this purpose. Many studies also focused on assessing *biomarkers*, i.e., biological markers associated with behavioral changes, that can predict or correlate with disease trajectories and treatment responses (Volkow et al., 2015; Campanella et al., 2020; Poireau et al., 2022). Nevertheless, despite all these efforts, current clinical outcomes remain modest (e.g., Fascher et al., 2024). This at least suggests that further studies should try to renew their approach of SUD clinical care (fostering for instance longer lengths of services; Beaulieu et al., 2021), and/or rely on refined SUD models (promoting for instance the inclusion of stress in neurocognitive models; Noël et al., 2013).

On this basis, an important role to be played by this section Addictive Behaviors in Frontiers in Psychology is to provide a platform for sharing and promoting the scientific outcomes of theoretical as well as empirical works highlighting the mechanisms triggering compulsive behaviors, elucidating protective as well as risky factors, or including new methods and/or experimental paradigms to face with the various forms of addictive behaviors. Importantly, the heterogeneous as well as (sometimes) the counter-intuitive data that SUD researchers have obtained so far clearly stresses the extreme complexity of this phenomenon. This should naturally push further studies to adopt a more *integrative perspective*, in which theoretical models would involve, in an interdisciplinary approach, social, behavioral, neural, emotional, cognitive and clinical manifestations of addictive behaviors as well as methodological parameters that better approximate the addictive experience, in order to help the field move toward improved understanding, prevention, diagnosis, and treatment (Diehl et al., 2018; Corley et al., 2024).
